# Experimental Study of the Dynamic and Static Compression Mechanical Properties of Closed-Cell PVC Foams

**DOI:** 10.3390/polym14173522

**Published:** 2022-08-27

**Authors:** Houqi Yao, Yuezhao Pang, Xin Liu, Jia Qu

**Affiliations:** Key Laboratory of Advanced Ship Materials and Mechanics, College of Aerospace and Civil Engineering, Harbin Engineering University, Harbin 150001, China

**Keywords:** PVC foams, SHPB, strain rate sensitivity

## Abstract

Closed-cell polyvinyl chloride foam (PVC) possesses many advantages, including its light weight, moisture protection, high specific strength, high specific stiffness, and low thermal conductivity, and is widely used as the core material in composite sandwich structures. It is increasingly used in fields with light weight requirements, such as shipbuilding and aerospace. Some of these structures can be affected by the action of dynamic loads during their lifespan, such as accidental or hostile blast loads as well as wind-loaded debris shocks. Examining the material properties of PVC foams under dynamic load is essential to predict the performance of foam sandwich designs. In this study, the compressive responses of a group of PVC foams with different densities were investigated under a broad range of quasi-static conditions and high strain rates using a universal testing machine and a lengthened Split Hopkinson press bar (SHPB) fabricated from titanium alloy. The results show that the mechanical properties of foam materials are related to their density and are strain rate-sensitive. The compressive strength and plateau stress of the foams were augmented with increased foam density. In the quasi-static strain rate range, the compressive strength of PVC foams at 10^−1^ s^−1^ was 27% higher than that at 10^−4^ s^−1^. With a strain rate of 1700 s^−1^, the strength was 107% higher than the quasi-static value at 10^−4^ s^−1^.

## 1. Introduction

Closed-cell foam is widely used in fields demanding high damage tolerance and lightweight materials [1,2]. For example, aluminum closed-cell foam is applied in the design of crash-proof beams in crash absorption boxes in vehicles [3,4], and due to their high strength and stiffness, foam sandwich composites have been widely applied in ship [5] and aircraft [6] structures.

Closed-cell polymer foams are particularly suited for structural applications due to their high compressive properties, enhanced dimensional stability, and high resistance to environmental corrosion. Several theoretical and experimental studies on closed-cell polymer foams have been published [7,8,9]. In recent years, closed-cell PVC foams have been widely applied in marine structures that are inevitably subject to various dynamic and complex loading effects during their lifespan [10,11]. In addition, the use of these structures on military ships places more stringent requirements on their life expectancy and stability; thus, it is necessary to evaluate the performance of the materials under different strain rate conditions.

Closed-cell PVC foam performance is mainly determined by the cell morphology, including the cell shape and size, cell wall thickness, and foam density [12,13]. Current research on various PVC foam preparation processes and designs is exhaustive [14], and the effects of processing parameters on foam structural morphology and properties have been thoroughly explored [15,16]. PVC plastic is a thermoplastic polymer containing both straight and branched chains that are interconnected by van der Waals forces. Through the addition of cross-linking agents, the chemical bonding between the linear chains can be promoted, thus improving their mechanical properties. The strain rate sensitivity in PVC foam mechanical properties is believed to be due to the viscoelasticity of PVC material. The Gibson Ashby model is widely used to describe the change trend in closed-cell foam mechanical properties and has been applied in PVC foam studies [9,17].

Several studies on the quasi-static (Q-S) or high strain rate (HSR) compressive properties of a variety of PVC foam compositions have been conducted. In the study by Luong et al. [18], closed-cell PVC foams were tested for dynamic compression properties at strain rates ranging from 0.0001 to 2773 s^−1^ using nylon SHPB. As a result of the experiment, PVC foam is sensitive to the strain rate. Thomas et al. [19] conducted compressive experiments on PVC foams with different densities by SHPB with polycarbonate bars. Their results show that the mechanical properties of PVC foams have a dependence on the temperature and strain rate. In a study by Mafhuz et al. [20,21], cross-linked PVC foams and PUR foams were studied under strain rates of 130/s and 1750/s by SHPB with polycarbonate bars. In the experiment, the specimen achieved equilibrium more quickly under lower strain rates when the stress equilibrium state was examined. There is a strong relationship between the foam density and strain rate, which determines the peak stress and energy absorption. According to Tagarielli et al. [22], compression tests were conducted on PVC foams with various densities at strain rates between 0.0001/s to 4000/s, and a linear relationship between compression and density was found. Daniel et al. [23,24] investigated the dynamic compressive properties of Divinycell H250 PVC foams. The range of strain rates is from 0.00002 to 1000 s^−1^. A linear relationship was found between the yield stress, peak stress, plateau stress, densification strain, and strain rate, although the modulus remained constant.

However, due to the variety of material compositions and test conditions used, from the available data, it is difficult to fully understand and determine closed-cell PVC foam mechanical properties and the relationship between the density and test strain rate. The present study aims to fill this critical gap by (1) investigating three types of foams made of the same material but with different densities and (2) characterising the selected foams over a broad range of compressive strain rates spanning seven orders of magnitude. The findings of this study can assist product designers in the rational selection of the materials required based on loading conditions and performance requirements.

## 2. Materials and Methods

### 2.1. Materials

Three types of closed-cell Divinycell PVC foams, supplied by DIAB, Inc., DeSoto, TX, were selected for the study. The nominal densities of these foams are 75, 160, and 250 kg/m^3^, which correspond to their nomenclature: H75, H160, and H250, respectively. The mechanical properties of these PVC foams, as provided by the manufacturer, are listed in Table 1 [25]. The foams are referred to as high-performance grade by the vendor, as they are designed to serve as sandwich core materials and to withstand the high temperatures and pressures encountered during sandwich fabrication.

### 2.2. Quasi-Static Compression Testing

Quasi-static compression tests were performed using an Instron 5500r electronic universal testing machine for quasi-static compressive experiments. A 10 KN load cell was used for the testing. The crosshead moving speed was controlled to achieve different quasi-static strain rate loadings on the samples using the testing machine supporting software. Cylindrical samples with a nominal diameter of 10 mm and height of 5 mm were used in the experiments. Samples were machined from the foam plates by a sculpting machine. Several previous studies have explored the anisotropy of PVC foam properties; therefore, all sample sampling and testing in this work were conducted solely in the thickness direction of the foam slab.

### 2.3. High Strain Rate Compression Testing

**Figure 1:**
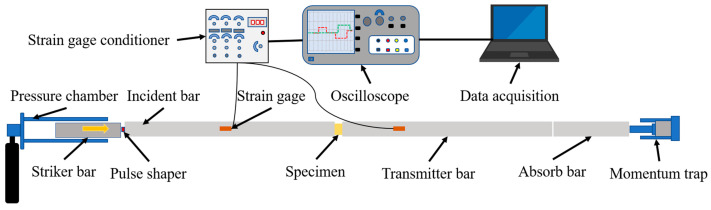
Schematic of the Split Hopkinson pressure bar set up for high strain rate testing.

The HSR compression tests were conducted using a Split Hopkinson pressure bar (SHPB) setup, schematically shown in Figure 1. Closed-cell PVC foam materials tend to be suitable for use in environments with lower strain rates and larger strains. To study the dynamic mechanical properties under such conditions, the samples were loaded with small amplitude and long wavelength stress pulses. Previous studies have generally found it difficult to obtain the complete wave shape during the compression of soft material samples, such as foams, due to the limitation of the rod length of conventional SHPB experimental devices. If the lengthening of the loading pulse length is achieved simply by lengthening the striker bar length, it will often lead to the incident and reflected waves interfering with each other through superimposition, making it difficult to obtain effective experimental data and increasing the difficulty of data processing. Meng and Q.M.L [26] used multi-group strain sheet pasting to separate the superimposed waveforms, but the strict requirements on the incident bar material and dimensions limited a wider range of applications of this method. In this paper, by employing a lengthened SHPB apparatus, we aimed to physically separate the incident and reflected waves. When testing soft materials such as PVC foams, bars of lower impedance materials such as aluminium [22,27], magnesium [20], polymers [28,29], or a combination of steel and plastic [30] have previously been used. In this study, the incident, transmitted, and striker bars of TC4 titanium alloy were used. The Young’s modulus and density of TC4 titanium alloy were taken as 110 GPa and 4510 kg/m^3^, respectively, in the calculation, and these properties were experimentally measured for the bars used in the experiment. To achieve stress balance by waveform shaping the incident wave, a silicone pulse shaping device was pasted on the end face, where the incident wave contacted the incident bar, to lengthen the stress pulse. A 2.5 m-length striker bar was used, and the length and diameter of the incident and transmitter bars were 8 m and 16 mm, respectively. A 1.5 m-long absorbing bar and momentum trap device were used to absorb the transmitter bar energy.

The test sample was clamped between the incident and transmitter bars of the SHPB apparatus, and the counter sample size was the same as that of the quasi-static sample used in the experiment. In the test, the strike bar impact velocity is achieved by releasing pressure stored in the pressure chamber. The striker bar is launched against the pulse charge to generate an incident wave, which propagates towards the incident bar and the sample. The incident wave, filtered by the pulse shaper, is sequentially transmitted to the incident bar, the sample, and the transmission bar. Due to the impedance mismatch between the sample and the incident bar, part of the incident wave is transmitted through the sample into the transmission bar (transmitted wave), and part of it is reflected back towards the impact end of the incident bar (reflected wave). The incident wave and the reflected wave are then taken from the strain gauge attached to the incident bar, whilst the transmitted wave is taken from the strain gauge attached to the transmission bar. The sample dynamic mechanical properties are then calculated using the above three waves. A change in the strain rate can be achieved by varying the velocity of the striker bar. The incident, reflected, and transmitted pulses were collected by two semiconductor strain gauges taped to the incident and transmitter bars, respectively, and then recorded by a Tektronix TDS 2000b oscilloscope after the strain plate signal was amplified by a strain gauge conditioner and stored by a data acquisition system.

### 2.4. Microstructural Observations and Imaging

The microstructure of the PVC foams was observed in pre-testing using HITACHI 3400n and 3500n scanning electron microscopes, samples were sprayed with gold-coated conductive layers, and optical micrographs were obtained using a Nikon EPIPHOT 200 optical microscope equipped with a Nikon DS-Fi 1 digital camera. The image acquisition and analysis were performed using NIS elements 3.0 software to measure the foam cell size and wall thickness.

## 3. Results and Discussion

### 3.1. Foam Microstructures

**Figure 2:**
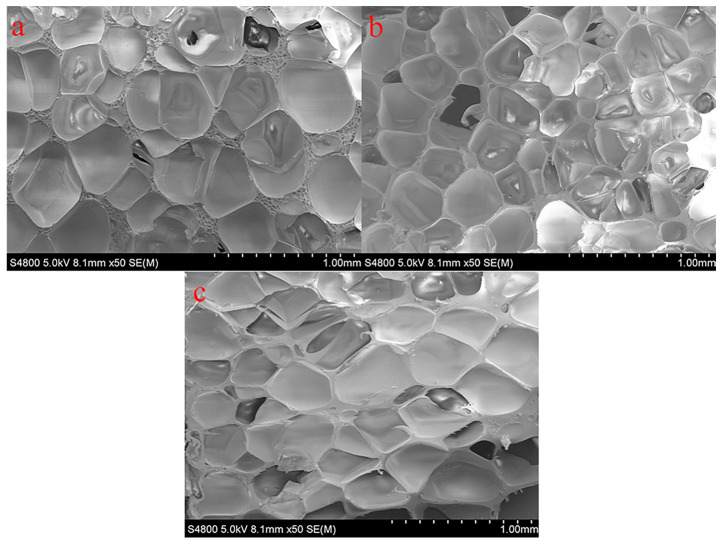
SEM image of as-received (**a**) H75, (**b**) H160, and (**c**) H250 PVC foams.

**Figure 3:**
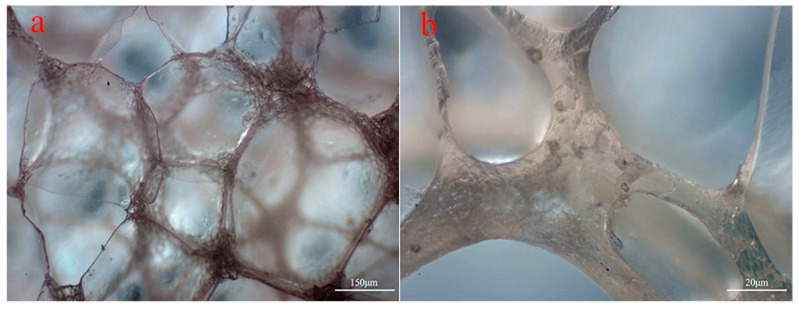
Optical micrographs of (**a**) H75 and (**b**) H250 foams.

**Figure 4:**
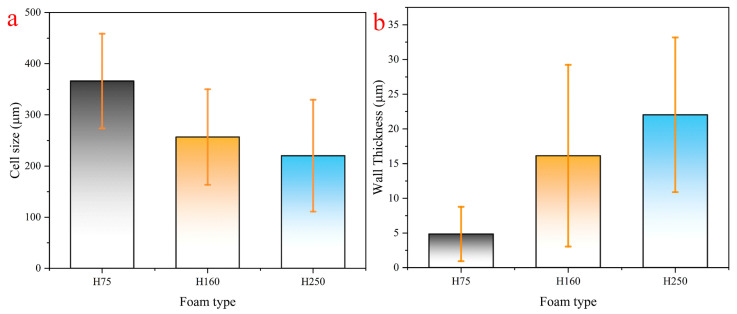
Measurements of PVC foam (**a**) cell size and (**b**) wall thickness.

The microstructures of the various types of PVC foams are shown in Figure 2. Foam samples were cut using sharp blades, and the microstructure of the cut surface is shown in these micrographs. All micrographs shown in Figure 2 were obtained at the same magnification to allow for a comparison of the microstructure of the individual foam types. Optical micrographs were used to conduct cell size and wall thickness analyses. Figure 3 shows the microstructure of foams under an optical microscope. It can be seen that the cell size and wall thickness vary greatly, even within the same sample type. Furthermore, the measurements were performed on a cutting surface, on which the largest cell size may not have fitted; therefore, due to the large variability in dimensions across the measurement plane, at least 100 cell dimension and 150 cell wall thickness measurements were taken from randomly selected areas. The measured parameters are plotted in Figure 4, where error bars represent the standard deviation. Since there is a significant difference in cell size, the standard deviation is larger when compared to the average vesicle size. However, a relationship was identified between the density of the foam samples and the cell size and wall thickness by observing the overall change trend in the mean values. As shown in Figure 4, the average H75 cell size is 40% larger—and the wall thickness is 80% smaller—than that of H250.

Figure 3b shows that changes in the foam cell wall thickness are characterised by the weakest point being at the centre of the vesicular pore and gradually increasing near multiple vesicular junctions. According to this foam microstructure characteristic, it can be determined that deformation and destruction will start at the weakest point in the foam structure under load. Although numerous studies have shown that foam strain rate sensitivity is related to multiple parameters of the foam material, such as the viscoelastic properties possessed by the polymer matrix itself, the foam microstructure characteristics are also one of the reasons for the high strain rate sensitivity of foam materials.

### 3.2. Foam Compressive Mechanical Response

**Figure 5:**
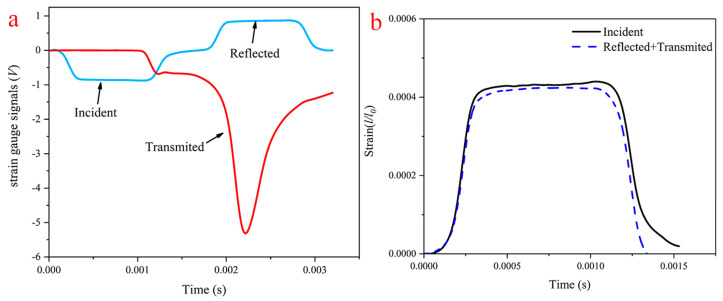
A representative set of (**a**) strain signals from incident and transmitter bars and (**b**) the stress equilibrium obtained in an H160 PVC foam specimen at a strain rate of 864 s^−1^.

The quasi-static compressive stress–strain curves of the foams were obtained from the displacement load data determined in the experiments via sample initial cross-sectional area and length calculations. SHPB impact compression experiments were able to derive the strain pulse signals from both incident and transmitter bars. A representative set of incident and transmitter bar strain signals obtained from an H75 PVC foam sample is plotted in Figure 5a. During impact compression, the incident wave forms as a rectangular wave, and the characteristics of the reflected and transmitted waves depend on the sample material. A lubricant is used between the sample and the bars to increase the contact surface area, reduce the friction effect on the end face, and facilitate the transfer of stress pulses through the interfaces. With the choice of a reasonable shaper device size, the samples were under stress equilibrium during the test, and the stress balance is shown in Figure 5b. In general, the stress balance conditions of polymeric foams in impact compression experiments are difficult to attain due to the non-uniform microstructure and high damping characteristics of the material. Since stress equilibrium was reached in the experiment, the strain rate, stress, and strain of the samples could be calculated by the following equation:

(1) $$\dot{\varepsilon }\left(t\right)=\frac{2c_{0}\varepsilon_{r}\left(t\right)}{l_{0}}$$

(2) $$\sigma \left(t\right)=\frac{AE\varepsilon_{t}\left(t\right)}{A_{0}}$$

(3) $$\varepsilon \left(t\right)=\int_{0}^{t}\dot{\varepsilon }\left(t\right)d\tau$$

where A and E are the cross-sectional area and Young’s modulus of the bar materials, respectively, and $c_{0}$ is the sound wave velocity in the bar. In addition, $l_{0}$ and $A_{0}$ are the length and cross-sectional area of the sample, respectively, $\varepsilon_{r}\left(t\right)$ and $\varepsilon_{t}\left(t\right)$ are the reflected and transmitted axial strain pulses with respect to time, respectively, and τ is a time variable used for integration.

**Figure 6:**
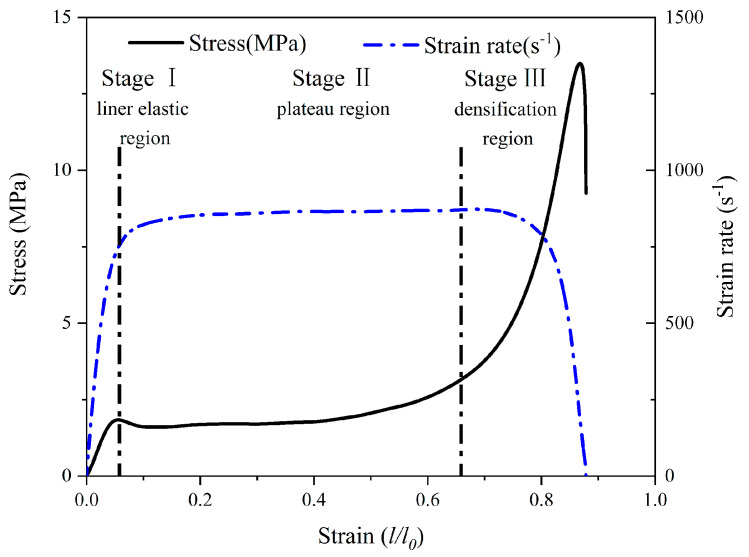
A representative set of stress–strain and strain rate–strain graphs of the H75 PVC foam test at 863.7 s^−1^.

Figure 6 shows a representative curve for a set of H75 PVC foams under a test condition strain rate of 864 s^−1^. The diagram divides the foam compressive stress–strain curve into three stages according to the changing characteristics of the curve under the foam compressive conditions. The first stage is the linear elastic stage of the foam, where the stress shows a linear increase, and the strain rate also increases sharply. In the second stage, the stress plateau stage, it is clear that the stress value changes slightly within a constant range, and the material strain continues to increase. Finally, in the third stage, the foam enters the densification stage due to most of the inner cell wall folding and collapsing under compression, and the internal stress increases sharply until the peak, whereupon it quickly decreases. The foam constant strain rate plateau region in the experiment played a decisive role in the foam’s response under high-strain-rate conditions, and the strain rate value in this study was calculated as the average of the strain rate plateau segment in stage II. In the current study, the PVC foam modulus is not reported, since, in stage I, where the modulus is calculated, the strain rate rapidly rises from 0 to over 800 s^−1^, and the measurements may be affected by the sharp strain rate change. This demonstrates the limitations of SHPB technology when processing material property characterisation.

**Figure 7:**
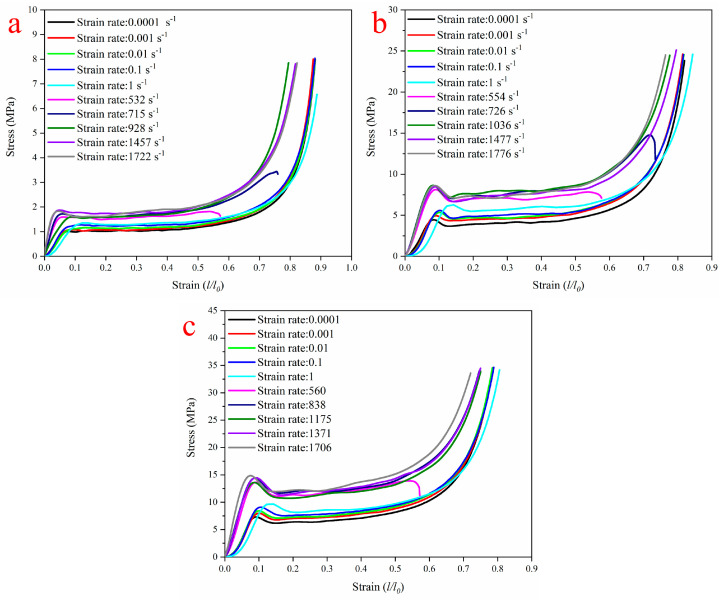
A representative of compressive stress–strain graphs for (**a**) H75, (**b**) H160, and (**c**) H250 at quasi-static conditions.

**Figure 8:**
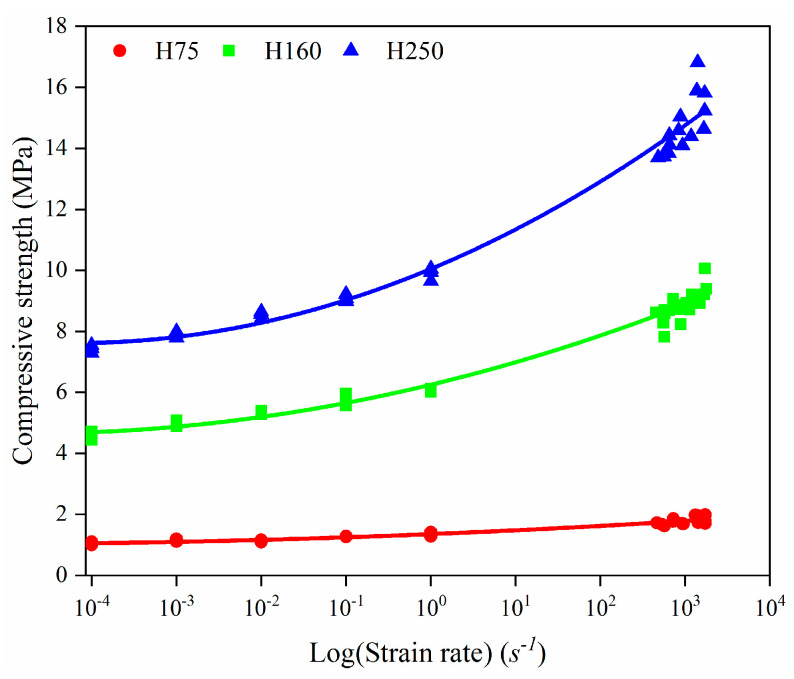
The compressive strength over the strain rate (log scale).

**Figure 9:**
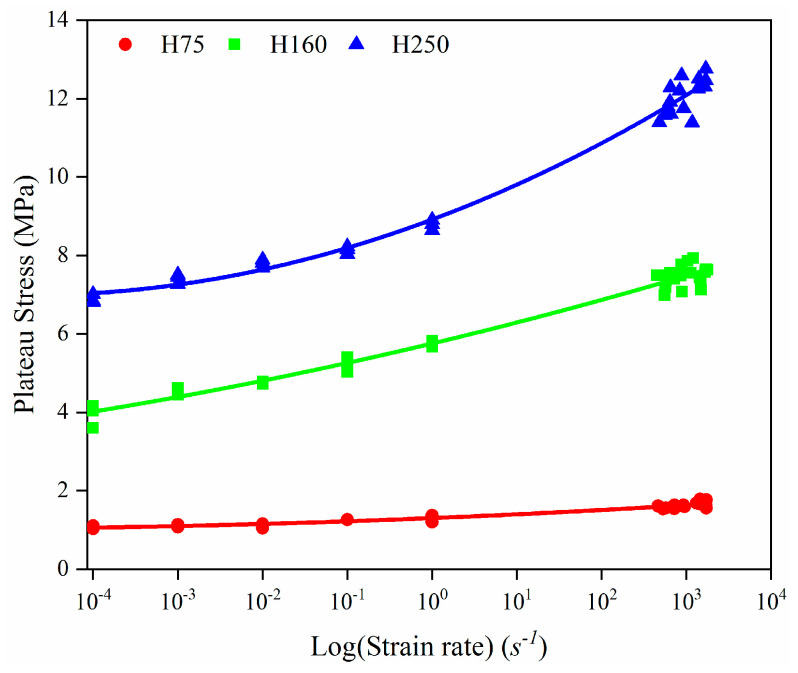
The plateau stress over the strain rate (log scale) for PVC foams.

The quasi-static and HSR compression stress–strain graphs for all three foam types are shown in Figure 7. As can be seen from the changing shape of the curves, the three types showed the same changing trend with most closed-cell porous materials under compression. Namely, the material in the initial stage is subjected to compressive load; then, the stress plateau phase occurs after the linear segment, corresponding to the deformation of the foam cell structure and the plastic deformation of the foam cell wall material. The densification stage follows the plateau stage, and the stress level increases sharply. From Figure 7, it can be seen that the curve of all specimens a with strain rate of about 550 s^−1^ is different from the other testing strain rate and shows no densification. This is due to the fact that soft material specimens typically develop a much larger deformation course in experiments, with maximum engineering strains that can often easily exceed 70%, and their required loading pulse widths can also reach up to 1 ms under normal experimental conditions, whereas the SHPB equipment used in this paper has a loading pulse duration limited to 900 μs. Consequently, when the strain rate is below 700 s^−1^, the curve contains only the linear elastic region and part of the plateau region. Under quasi-static and lower-strain-rate conditions, there are no evident fracture points in the samples, even with 50% or more strains. For porous material foam, the first stress peak occurring after the end of the linear stage is defined as the foam compressive strength, and the stress level on the stress plateau is defined as the plateau stress. The compressive strength and plateau stress versus the strain rate for different foam types are shown in Figure 8 and Figure 9. Generally, the foam compressive deformation plateau phase is not a constant value and fluctuates constantly within a small range, and the plateau stress is the average value of this phase.

It can be seen from Figure 7 that both the foam compressive strength and plateau stress improve with the increased strain rate. Relative to the quasi-static experimental conditions, the compressive strength of H75, H160, and H250 PVC foams under high-strain-rate conditions increased by 98%, 110%, and 113%, respectively, and the plateau stress increased by 70%, 89%, and 87%, respectively. As an energy-absorbing material, the total energy absorption within the foam can be increased by either increasing the plateau stress or the densification strain.

The dependence of peak stress (σ(ε)) on the compressive strain rate ($\dot{\varepsilon }$) is defined by a power law relation [30]:

(4) $$\frac{\sigma \left(\varepsilon \right)}{\sigma_{0}\left(\varepsilon \right)}={\left(\frac{\dot{\varepsilon }}{{\dot{\varepsilon }}_{0}}\right)}^{m}$$

where ${\dot{\varepsilon }}_{0}$ is the reference strain rate, $\sigma_{0}\left(\varepsilon \right)$ is the reference stress, and m is the power law exponent. Considering the reference stress and reference strain rate as 1, the calculated values for the power law exponent of compressive strength and plateau stress are presented in Table 2. The results presented in Figure 8 and Figure 9 show that H75 PVC foams exhibited negligible strain rate effects in the 10^−4^–10^−2^ s^−1^ strain rate range but presented a clear power exponential strain rate sensitivity at higher strain rates. Whereas H160 and H250 PVC foams exhibited a pronounced strain rate sensitivity at low-strain-rate conditions, the strain rate sensitivity was amplified with increasing foam density. From the results of Table 2, it can be found that the range of change in the sum values obtained in this study is similar to the results reported for various closed-cell PVC foams in a concurrent study [31].

**Figure 10:**
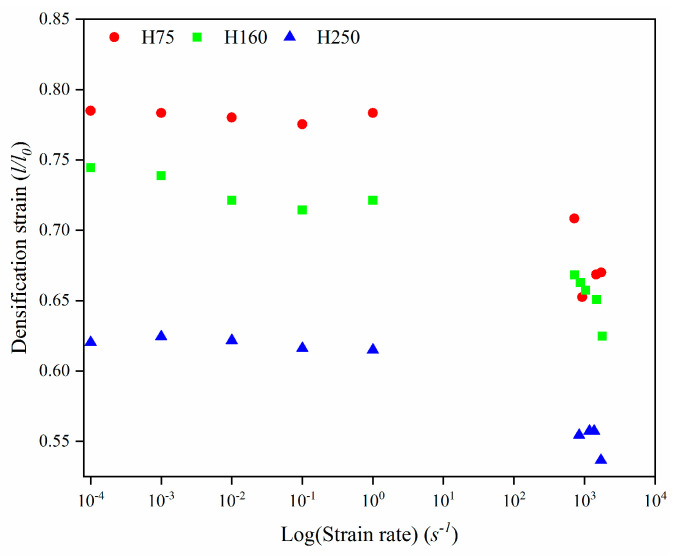
The densification strain over the strain rate (log scale) of PVC foams.

To explore the relationship between the foam density and densification strain, the sample densification strain is calculated by the tangent intersection points drawn in the plateau region and the densification region of the stress–strain graphs, as described by Mondal et al. [31]. Figure 10 depicts the material densification strain values as a function of foam density and strain rate.

The foams tested using SHPB at strain rates below 700 s^−1^ did not attain the densification strain; therefore, the data from those samples are not included in Figure 10. It can be seen that the densification strain values of the higher density foams are lower due to their lower porosity and greater wall thickness. The densification strains of the three dense PVC foams under high-strain-rate experimental conditions all reduced by various degrees, and under quasi-static conditions, the densification strains of the H75, H160, and H250 PVC foams under high-strain-rate conditions were reduced by 17%, 16%, and 14%, respectively.

## 4. Conclusions

In this paper, the compressive mechanical responses of three different density PVC foams were experimentally investigated, under dynamic and static conditions, using a universal testing machine and SHPB apparatus. To obtain the complete waveform of the PVC foams under high-strain-rate conditions, a long titanium alloy SHPB device was used, and an appropriately sized waveform former was selected to achieve constant high-strain-rate loading on the samples. In addition, a semiconductor strain gauge capable of detecting low stresses in soft materials at strain rates in the range of 500–1700 s^−1^ was used to measure the rod strain.

The results revealed that the compressive mechanical response of PVC foams is a strain rate sensitivity in the range of 1 × 10^−4^–2 × 10^3^, which increased with higher foam densities.

The compressive strength and plateau stress of foams depend on the foam density; therefore, higher density foams have superior mechanical properties. Compressive strength and plateau stress intensity depend on the quasi-static strength of the foam and the magnitude of the compressive strain rate under high-strain-rate conditions. For the H75, H160, and H250 PVC foams, the plateau strength increased by more than 60% with respect to the static strength at strain rates of the order of 10^3^ s^−1^.

## Figures and Tables

**Table 1:** Parameters of PVC foams [25].

Property	Unit	H180	H160	H250
Plastic stress	MPa	1.4	3.4	7.2
Compressive modulus	MPa	90	200	4000
Tensile strength	MPa	3.5	5.4	9.2
Tensile modulus	MPa	95	205	320
Shear strength	MPa	1.15	2.6	4.5
Shear modulus	MPa	27	60	97
Poisson ratio	MPa	-	0.4	0.4

**Table 2:** Power law exponent for various PVC foams.

Foam Type	Compression Strength		Plateau Stress	
	*σ*_0_ (MPa)	*m*	*σ*_0_ (MPa)	*m*
H75	1.36	0.038	1.30	0.031
H160	6.09	0.052	5.73	0.039
H250	9.89	0.055	8.78	0.043

## Data Availability

Not applicable.

## References

[1] Al-Shamary A.K.J., Karakuzu R., Özdemir O. (2016). Low-velocity impact response of sandwich composites with different foam core configurations. J. Sandw. Struct. Mater..

[2] Feng D., Aymerich F. (2020). Effect of core density on the low-velocity impact response of foam-based sandwich composites. Compos. Struct..

[3] Mohammadiha O., Beheshti H., Aboutalebi F.H. (2015). Multi-objective optimisation of functionally graded honeycomb filled crash boxes under oblique impact loading. Int. J. Crashworthiness.

[4] Abdullah N., Sani M., Salwani M., Husain N. (2020). A review on crashworthiness studies of crash box structure. Thin-Walled Struct..

[5] Zhang S., Villavicencio R., Zhu L., Pedersen P.T. (2017). Impact mechanics of ship collisions and validations with experimental results. Mar. Struct..

[6] Marx J.C., Robbins S.J., Grady Z.A., Palmieri F.L., Wohl C.J., Rabiei A. (2020). Polymer infused composite metal foam as a potential aircraft leading edge material. Appl. Surf. Sci..

[7] Christensen R.M. (2000). Mechanics of cellular and other low-density materials. Int. J. Solids Struct..

[8] Torquato S., Gibiansky L., Silva M., Gibson L. (1998). Effective mechanical and transport properties of cellular solids. Int. J. Mech. Sci..

[9] Mills N. (2007). Polymer Foams Handbook: Engineering and Biomechanics Applications and Design Guide.

[10] Sutherland L.S. (2018). A review of impact testing on marine composite materials: Part II—Impact event and material parameters. Compos. Struct..

[11] Sutherland L.S. (2018). A review of impact testing on marine composite materials: Part III—Damage tolerance and durability. Compos. Struct..

[12] Köll J., Hallström S. Morphology effects on constitutive properties of foams. Proceedings of the 18th International Conference on Composite Materials.

[13] Colloca M., Dorogokupets G., Gupta N., Porfiri M. (2012). Mechanical properties and failure mechanisms of closed-cell PVC foams. Int. J. Crashworthiness.

[14] Grossman R.F. (2008). Handbook of Vinyl Formulating.

[15] Wu Q., Zhou N., Zhan D. (2009). Effect of processing parameters and vibrating field on poly (vinyl chloride) microcellular foam morphology. Polym. -Plast. Technol. Eng..

[16] Jin F.-L., Zhao M., Park M., Park S.-J. (2019). Recent trends of foaming in polymer processing: A review. Polymers.

[17] Altenbach H., Eremeyev V.A. (2010). Thin-walled structures made of foams. Cellular and Porous Materials in Structures and Processes.

[18] Luong D.D., Pinisetty D., Gupta N. (2013). Compressive properties of closed-cell polyvinyl chloride foams at low and high strain rates: Experimental investigation and critical review of state of the art. Compos. Part B Eng..

[19] Thomas T., Mahfuz H., Carlsson L.A., Kanny K., Jeelani S. (2002). Dynamic compression of cellular cores: Temperature and strain rate effects. Compos. Struct..

[20] Chakravarty U., Mahfuz H., Saha M., Jeelani S. (2003). Strain rate effects on sandwich core materials: An experimental and analytical investigation. Acta Mater..

[21] Saha M., Mahfuz H., Chakravarty U., Uddin M., Kabir M.E., Jeelani S. (2005). Effect of density, microstructure, and strain rate on compression behavior of polymeric foams. Mater. Sci. Eng. A.

[22] Tagarielli V., Deshpande V., Fleck N.A. (2008). The high strain rate response of PVC foams and end-grain balsa wood. Compos. Part B Eng..

[23] Daniel I.M., Cho J.-M. (2011). Characterization of anisotropic polymeric foam under static and dynamic loading. Exp. Mech..

[24] Daniel I.M., Cho J.-M., Werner B.T. (2013). Characterization and modeling of stain-rate-dependent behavior of polymeric foams. Compos. Part A Appl. Sci. Manuf..

[25] DIAB (2017). Divinycell H Technical Manual.

[26] Meng H., Li Q. (2003). An SHPB set-up with reduced time-shift and pressure bar length. Int. J. Impact Eng..

[27] Chen W., Lu F., Frew D.J., Forrestal M.J. (2002). Dynamic compression testing of soft materials. J. Appl. Mech..

[28] Ouellet S., Cronin D., Worswick M. (2006). Compressive response of polymeric foams under quasi-static, medium and high strain rate conditions. Polym. Test..

[29] Mahfuz H., Al Mamun W., Haque A., Turner S., Mohamed H., Jeelani S. (2000). An innovative technique for measuring the high strain rate response of sandwich composites. Compos. Struct..

[30] Nagy A., Ko W., Lindholm U. (1974). Mechanical behavior of foamed materials under dynamic compression. J. Cell. Plast..

[31] Mondal D., Goel M., Das S. (2009). Compressive deformation and energy absorption characteristics of closed cell aluminum-fly ash particle composite foam. Mater. Sci. Eng. A.

